# Changes in Active Commuting to School in Czech Adolescents in Different Types of Built Environment across a 10-Year Period

**DOI:** 10.3390/ijerph121012988

**Published:** 2015-10-16

**Authors:** Jan Dygrýn, Josef Mitáš, Aleš Gába, Lukáš Rubín, Karel Frömel

**Affiliations:** Faculty of Physical Culture, Palacký University, Olomouc 771 11, Czech Republic; E-Mails: josef.mitas@upol.cz (J.M.); ales.gaba@upol.cz (A.G.); lukas.rubin@upol.cz (L.R.); karel.fromel@upol.cz (K.F.)

**Keywords:** walking, cycling, physical activity, GIS, youth, transportation, census

## Abstract

Active commuting (AC) to school represents a great opportunity to incorporate walking or cycling into adolescents’ everyday routine. The objective of the study was to describe changes in AC in Czech adolescents across a 10-year period in different built environments. Data from the 2001 and 2011 Czech Census of Population and Housing were used to examine the mode of transportation taken to school in 6236 adolescents. Changes in AC over time were analyzed for low and high walkable areas separately in two Czech regional cities, Olomouc and Hradec Králové. Between 2001 and 2011, the proportion of adolescents actively commuting to school decreased by 47%, from an absolute rate of 49.1% to 26%. The proportion of active commuters fell in low walkable areas by 61% and in high walkable areas by 39%. The results indicated that adolescents in 2011 were 2.7 times less (OR = 0.365, *p* < 0.001) likely to actively commute than in 2001. The AC behavior in Czech adolescents has a negative tendency to replicate travel-to-school patterns in adolescents previously described in more developed countries. The findings might serve as a recommendation for municipal policy.

## 1. Introduction

Being physically active is one of the most important steps that people of all ages can take to improve their health [1]. Active commuting (AC) is defined as walking, cycling, or other forms of human-powered transport used for utilitarian purposes, e.g., transporting to work or school. [2]. Regular walking or cycling to school or work might positively affect human health, and lead specifically to body weight reduction [3] and improved cardiovascular risk profiles in children and young people [4]. AC also represents a great opportunity to incorporate walking or cycling into adolescents’ everyday routine and can represent 20% to 30% of daily moderate-to-vigorous physical activity [5]. It is necessary to monitor and understand the trends and changes in AC because changing the mode of transport from motorized to non-motorized results in a significant decrease in local carbon dioxide emissions and noise, and other negative effects associated with the use of passenger cars [6]. In the area of sustainable development, AC is understood as one of the real ways of decreasing the human carbon trace, decreasing the use of motorized transport, and thereby energy consumption [7].

To successfully promote AC it is important to understand the factors that influence AC. Current theoretical models indicate that, similar to complex human behavior, an individual’s choice to use an active mode of transport is influenced by various factors. The most significant factors include personality characteristics (e.g., perception of barriers to AC), social environment (e.g., family support), public policy (e.g., subsidies for bike hire, discount on value-added tax), and the built environment (e.g., aesthetics, residential density, land use mix, car-free zones). In particular, environmental conditions appear to be one of the most important elements in AC behavior. Although numerous studies examined environmental correlates (e.g., residential density, connectivity, and land use mix) of active commuting in adults [8,9,10], there is limited evidence of studies focused on adolescents. The relationships between residential density, connectivity, and land use mix (attributes used to define the walkability of areas) and active transport in adolescents are still not well understood. Kerr [11] found that a greater walkability of a neighborhood was associated with more active commuting in respondents aged 5–18 years. However, findings for the association of active commuting with land use mix, street connectivity, and presence of busy road have been mixed [12,13,14,15]. High land use mix around the place of residence and high connectivity facilitates zone permeability. Residential density expresses potential activity or dynamics of the area. These environmental attributes are used to define the walkability of areas [16,17,18]. Among Czech youth, the period between 2002 and 2010 was characterized by an increasing percentage of overweight or obese individuals, increased sedentary time, and decline or stagnation of the proportion of children meeting physical activity recommendations [19]; a similar pattern can be expected in AC. To our knowledge, there is no study using census data across a 10-year period to observe changes in adolescents’ AC in the Czech Republic. Therefore, the objective of the present study was to describe the changes in AC among adolescents in two Czech cities across a 10-year period by objectively measuring built environment and sex.

## 2. Materials and Methods

### 2.1. Procedures

The study was conducted in Olomouc and Hradec Králové, *i.e.*, cities where built environments have been assessed in the long term as a part of the International Physical Activity and the Environment Network (IPEN) project [18,20]. Olomouc is the sixth largest city in the Czech Republic with approximately 100,000 inhabitants and a population density of 987 inhabitants per square kilometer; Hradec Králové is the eighth largest city with approximately 93,000 inhabitants and a population density of 878 inhabitants per square kilometer. As a comparison, the population density of Prague (the capital city with more than 1.2 million residents) is 2539 inhabitants per square kilometer. Data collection took place in March 2001 and 2011.

Anonymous travel-to-school data from the 2001 and 2011 Czech Census of Population and Housing were obtained from the Czech Statistical Office for the purposes of scientific research. The census covered all permanent residents on the territory of the Czech Republic at the moment of the census and all persons having long-term residence in the Czech Republic at the moment of the census.

The dataset included all adolescents aged 12–17 whose permanent residence was in Olomouc or Hradec Králové. The legal guardian of a minor child provided the travel-to-school data. The exact wording of the question is as follows: “Specify the mode/s of transport that you usually use on a single way to work or school. Select from the below: Bus (except city transport), city transport, car-driver, car-passenger, train, bicycle, motorcycle, other, none (only walking)”. The sample from 2001 included 3164 adolescents and, in 2011, data from 3072 adolescents were eligible for the analysis. 

### 2.2. Active Commuting

For the purposes of the study, AC is understood as commuting by walking (no means of commuting used) or cycling; thus, people who used multiple modes of transport were excluded from the analysis (most notably walking combined with public transport). Therefore, these results under-represent the total number of adolescents who walk or cycle as a part of their journey to school.

### 2.3. Built Environment

Geographic information systems (GIS) were used to analyze and calculate the walkability indexes. Most studies assess walkability according to street connectivity, residential density, and land use mix, which experts believe to be most influential on physical activity and particularly on AC. The walkability index was adapted from international studies [11,20] and calculated separately for 2001 and 2011. Details on the index are described elsewhere [16]. Walkability was defined for each census block in Olomouc and Hradec Králové. The index was calculated using the following equation: Walkability = (z-score connectivity + z-score net residential density + z-score land use mix). Connectivity was defined as the number of intersections with three or more intersecting streets per square kilometer. Residential density was assessed as the number of residential units per square meter of residential area. Land use mix was assessed as evenness of distribution of building floor area of residential, commercial, institutional, and recreational development. According to the results of the walkability index, the census blocks were ranked and divided into deciles. The bottom four (1–4) deciles represented low and the top four (7–10) deciles represented high walkability areas. The fifth and sixth deciles were omitted from the analysis to create a separation between low and high walkability environments. The walkability index (2011) values ranged from −5.39 to 14.62 in Olomouc, and from −3.49 to 12.17 in Hradec Králové. The fact that two regions with completely different sources of data produced similar walkability values supports the face validity of the index. All calculations of the walkability index and all map layers were processed using ArcGIS software, version 10 (ESRI Inc., Redlands, CA, USA).

### 2.4. Statistical Analysis

The data analyses were conducted using IBM SPSS, version 19 (SPSS for Windows; SPSS, Chicago, IL, USA). Descriptive statistics are presented as mean and standard deviations. All analyses were performed with alpha set at 0.05.

The changes in AC over time were analyzed by sex and for low and high walkable areas separately. Logistic regression was used to evaluate a 10-year change in AC. The AC was the dependent variable with the year of survey as the independent categorical variable; 2001 was considered the reference year. The results of a regression analysis are presented as odds ratio (OR) with a 95% confidence interval. Change over time was calculated by dividing the difference at the two time points by the earlier time point.

## 3. Results

The analysis included data from 6236 adolescents, of which 50.1% were boys and 49.9% were girls, aged 12 to 17 years, with a mean age of 14.7 ± 1.7 years. In the monitored period there was a decrease in the number of adolescents walking or biking to school. Detailed information about the study sample is shown in Table 1.

**Table 1:** Descriptive statistic of study samples.

Attribute	2001		2011		2001 *vs.* 2011
	*N*	%	*N*	%	∆ ^a^
**Age categories**					
12–14 years	1656	52.3	1233	40.1	−23%
15–17 years	1508	47.7	1839	59.9	26%
Total sample	3164	100	3072	100	
**Mode of transport**					
Only walking	1466	46.3	769	25.0	−46%
Bicycle	88	2.8	29	0.9	−68%
City transport	1468	46.4	1683	54.8	18%
Bus	73	2.3	258	8.4	265%
Car-passenger	28	0.9	110	3.6	300%
Others	41	1.3	223	7.3	462%
Total sample	3164	100	3072	100	

**^a^** Relative change in proportion of respondents between 2001 and 2011.

Between 2001 and 2011, there was a change of the built environment related to the walkability of the area. In Olomouc, residential density decreased by 9%, respectively, from 5602.3 in 2001 to 5092.8 person/km^2^ in 2011. Connectivity decreased by 10%, respectively, from 48.2 to 43.6 intersection/km^2^. In Hradec Králové, residential density decreased by 3%, respectively, from 4481.6 in 2001 to 4351.5 person/km^2^ in 2011. Connectivity decreased by 11%, respectively, from 36.4 to 32.5 intersection/km^2^.

During the monitored period, in both low and high walkable areas, we observed a decrease in the proportion of adolescents actively commuting (combined walking and cycling) to school by 47% (relative decrease), from an absolute rate of 49.1% to 26.0% (Table 2). The proportion of active commuters fell by 61% in low walkable areas (from 37.4% to 14.6%) and in high walkable areas by 39% (from 54.5% to 33.2%). The logistic regression showed that adolescents in 2011 were 2.7 times less likely (OR = 0.365, *p* < 0.001) to actively commute than in 2001. Adolescents living in low walkable areas were 3.5 less likely (OR = 0.285, *p* < 0.001) to actively commute than in 2001 and those living in high walkable areas were 2.4 times less likely (OR = 0.416, *p* < 0.001) to actively commute in 2011 than in 2001. The highest decrease in AC was found identically in boys and girls from low walkable areas, and the lowest decrease was found in boys from high walkable areas. There were no differences in changes of AC by sex.

**Table 2:** Changes in active commuting to school between 2001 and 2011 by sex and type of walkable area.

Type of WA	2001		2011		2001 *vs.* 2011			
	*N*	% of AC	*N*	% of AC	∆ ^a^	OR ^b^	95% CI	*p*-Value
**Boys**								
Low	508	38.8	599	15.0	−61%	0.279	0.210–0.372	<0.001
High	1079	55.8	936	36.0	−35%	0.446	0.373–0.533	<0.001
All areas	1587	50.3	1535	27.8	−45%	0.380	0.328–0.441	<0.001
**Girls**								
Low	489	36.0	596	14.1	−61%	0.292	0.217–0.392	<0.001
High	1088	53.2	941	30.5	−43%	0.386	0.321–0.463	<0.001
All areas	1577	47.9	1537	24.1	−50%	0.346	0.297–0.404	<0.001
**Total sample**								
Low	997	37.4	1 195	14.6	−61%	0.285	0.232–0.350	<0.001
High	2167	54.5	1877	33.2	−39%	0.416	0.366–0.473	<0.001
All areas	3164	49.1	3072	26.0	−47%	0.365	0.327–0.404	<0.001

AC, active commuting; CI, confidence interval; OR, odds ratio; WA, walkable area; **^a^** Relative difference in percentage of actively commuting between 2011 and 2001; **^b^** Cohort from year 2001 is a reference group.

Between 2001 and 2011, the proportion of adolescents walking to school decreased by 46% (relative decrease), from an absolute rate of 46.3% to 25.0% (Table 3). In low walkable areas the decline was 60% (from 34.6% to 13.7%), and in high walkable areas the decline was 38% (from 51.7% to 32.2%). The results of logistic regression showed that, in 2011, adolescents were less likely (OR = 0.387, *p* < 0.001) to walk to school than those in 2001. Adolescents living in low walkable areas were less likely (OR = 0.301, *p* < 0.001) to walk to school in 2011 than those in 2001 and, in 2011, those living in high walkable areas were less likely (OR = 0.444, *p* < 0.001) to walk to school than adolescents in 2001. The highest decrease in relative change (61%) of walking to school between 2001 and 2011 was found in boys from low walkable areas, and the lowest change (33%) was found in boys from high walkable areas.

**Table 3:** Changes in walking to school between 2001 and 2011 by sex and type of walkable area.

Type of WA	2001		2011		2001 *vs.* 2011			
	*N*	% of Walkers	*N*	% of Walkers	∆ ^a^	OR ^b^	95% CI	*p*-Value
**Boys**								
Low	508	34.4	599	13.5	−61%	0.298	0.221–0.401	<0.001
High	1079	51.0	936	34.3	−33%	0.502	0.419–0.601	<0.001
All areas	1587	45.7	1535	26.2	−43%	0.422	0.363–0.490	<0.001
**Girls**								
Low	489	34.8	596	13.9	−60%	0.304	0.226–0.409	<0.001
High	1088	52.5	941	30.2	−42%	0.391	0.326–0.470	<0.001
All areas	1577	47.0	1537	23.9	−49%	0.354	0.304–0.413	<0.001
**Total sample**								
Low	997	34.6	1195	13.7	−60%	0.301	0.244–0.371	<0.001
High	2167	51.7	1877	32.2	−38%	0.444	0.390–0.505	<0.001
All areas	3164	46.3	3072	25.0	−46%	0.387	0.347–0.431	<0.001

CI, confidence interval; OR, odds ratio; WA, walkable area; **^a^** Relative difference in percentage of actively commuting between 2011 and 2001; **^b^** Cohort from year 2001 is a reference group.

A similar pattern occurred for cycling to school with a relative decrease by 68% and 71%. respectively. in low walkable areas and by 64% in high walkable areas (Table 4). The highest decrease in relative change (83%) of cycling to school between 2001 and 2011 was found in girls from low walkable areas, and the lowest change (57%) was found in girls from high walkable areas. The results of logistic regression showed that in 2011 adolescents were less likely (OR = 0.333, *p* < 0.001) to cycle to school than in 2001.

**Table 4:** Changes in cycling to school between 2001 and 2011 by sex and type of walkable area.

Type of WA	2001		2011		2001 *vs.* 2011			
	*N*	% of Cyclists	*N*	% of Cyclists	∆ ^a^	OR ^b^	95% CI	*p*-Value
**Boys**								
Low	508	4.3	599	1.5	−65%	0.337	0.154–0.739	0.007
High	1079	4.8	936	1.7	−65%	0.343	0.195–0.606	<0.001
All areas	1587	4.7	1535	1.6	−66%	0.339	0.214–0.536	<0.001
**Girls**								
Low	489	1.2	596	0.2	−83%	0.432	0.114–1.632	0.216
High	1088	0.7	941	0.3	−57%	0.135	0.016–1.128	0.064
All areas	1577	0.9	1537	0.3	−67%	0.291	0.096–0.887	0.030
**Total sample**								
Low	997	2.8	1195	0.8	−71%	0.292	0.141–0.604	0.001
High	2167	2.8	1877	1.0	−64%	0.359	0.214–0.604	<0.001
All areas	3164	2.8	3072	0.9	−68%	0.333	0.218–0.508	<0.001

CI, confidence interval; OR, odds ratio; WA, walkable area; **^a^** Relative difference in percentage of actively commuting between 2011 and 2001; **^b^** Cohort from year 2001 is a reference group.

## 4. Discussion 

The results of the present study indicate that the proportion of adolescents walking or cycling to school decreased between 2001 and 2011. A relative as well as an absolute increase in the number of adolescents using the passive mode of transport to school were observed for city transport, bus, and car-passenger. This finding is in line with a previous study of Czech youth pointing to increase of sedentary time and a decline or stagnation of the proportion of children meeting physical activity recommendations [19]. According to another study [21], a substantial part of Czech boys and girls (aged 11–15) do not participate in moderate-to-vigorous physical activity and vigorous physical activity as recommended. This decrease in AC might be associated with the social and economic transformation of Czech society and the fact that lifestyle changes have a tendency to replicate travel-to-school patterns that have been previously observed in the U.S. [22], Australia [23], Canada [24], the UK [25], and Spain [26].

Our results provide evidence that a proportion of adolescents walking or cycling to school is indeed linked to neighborhood environment characteristics. These findings are in agreement with other studies [27,28,29]. The decline of active commuters was stronger in low walkable areas than in high walkable areas. The findings suggest that the type of built environment influences the prevalence of AC to school and even influences the degree of decrease over time. Similar patterns were observed for both walking and cycling. Our findings may be explained by proximity to school in high walkable areas and by the fact that parents who live in high walkable areas are less likely to use cars (short distances between places and good access to public transport).

Consistent with previously published studies [29,30,31], walking was the most common mode of AC to school. On the other hand, in Belgium, where 58.4% of adolescents commuted actively to school, 88.7% of these active commuters cycled to school and 11.3% walked to school [32]. Studies examining barriers to AC have identified the distance between destinations and street safety as an important parental concern [11,33]. For walking-only journeys, the generally considered distance is approximately 2 km [34,35]. Our data analysis did not include distance variables and this limitation must be taken into account while interpreting the results. We demonstrated no sex differences in AC to school of Czech adolescents. This results are consistent with previous Czech research of AC of adolescents [36], but inconsistent with the results that have been reported in AC among boys and girls in the U.S. [23], Australia [23], Canada [24], the UK [25], and Spain [26].

There were some limitations in our study. The national census is one of the most extensive statistical surveys. It provides a great number of valuable data which cannot be effectively collected in any other way. However, the census does not ask about walking or cycling for recreation or sport and therefore underestimates the total walking and cycling. It is necessary to mention that AC is strongly affected by seasonality. For our purposes we excluded all journeys with more than one mode of transport from active transport, which led to underestimation of the total transportation of walking or cycling. Another limitation might be the fact that we are not able to verify whether the decrease in AC between 2001 and 2011 could be influenced by extended distances between schools and homes of adolescents. The changes in AC of adolescents may be also influenced by intense use of cars by adults, because there was a 41% increase (from 345 to 485 cars) in the number of automobiles per 1000 inhabitants between 2001 and 2011. Changes in attributes of the built environment not observed in our study could have happened during the 10-year period of 2001–2011, which also might be the source of the decrease in AC. A strength of the study is the large sample from the census data. Another strength is the application of objective assessment of environmental characteristics using the GIS separately for 2001 and 2011.

## 5. Conclusions

This study demonstrate that adolescents’ AC to school significantly decreased between 2001 and 2011 and was influenced by the built environment. We found that AC behavior of Czech adolescents has a tendency to replicate travel-to-school patterns of adolescents that have been previously observed in more developed countries. This finding has implications for the broader research community and it might serve as a recommendation for municipal policy. Increasing AC can have positive benefits for communities and it can also improve the health of individuals.
